# Tumor-Associated Lymphatic Vessel Features and Immunomodulatory Functions

**DOI:** 10.3389/fimmu.2019.00720

**Published:** 2019-04-09

**Authors:** Laure Garnier, Anastasia-Olga Gkountidi, Stephanie Hugues

**Affiliations:** Department of Pathology and Immunology, Faculty of Medicine, University of Geneva, Geneva, Switzerland

**Keywords:** lymphatic vessels, anti-tumor immune response, metastasis, lymphangiogenesis, tumor microenvironment

## Abstract

The lymphatic system comprises a network of lymphoid tissues and vessels that drains the extracellular compartment of most tissues. During tumor development, lymphatic endothelial cells (LECs) substantially expand in response to VEGFR-3 engagement by VEGF-C produced in the tumor microenvironment, a process known as tumor-associated lymphangiogenesis. Lymphatic drainage from the tumor to the draining lymph nodes consequently increases, powering interstitial flow in the tumor stroma. The ability of a tumor to induce and activate lymphatic growth has been positively correlated with metastasis. Much effort has been made to identify genes responsible for tumor-associated lymphangiogenesis. Inhibition of lymphangiogenesis with soluble VEGFR-3 or with specific monoclonal antibodies decreases tumor spread to LNs in rodent models. Importantly, tumor-associated lymphatics do not only operate as tumor cell transporters but also play critical roles in anti-tumor immunity. Therefore, metastatic as well as primary tumor progression can be affected by manipulating tumor-associated lymphatic remodeling or function. Here, we review and discuss our current knowledge on the contribution of LECs immersed in the tumor microenvironment as immunoregulators, as well as a possible functional remodeling of LECs subsets depending on the organ microenvironment.

## Introduction

Over the last few years, immunotherapy has evolved into a very promising new approach for fighting tumor progression. However, the proportion of cancer patients that positively respond to these treatments is still limited. Indeed, tumor cells foster mechanisms to escape immunosurveillance either by inducing poorly immunogenic tumors (immunoselection) or by setting up a tolerogenic environment that inhibits immune effector cells (immunosubversion) [reviewed in (1–3)]. Therefore, manipulations aiming at boosting anti-tumor immune cell responses and in particular tumor-specific T cell priming currently represent an extensive axis of investigations.

During tumor development, lymphatic endothelial cells (LECs), the principal components of lymphatic vessels (LVs), undergo active modifications that facilitate metastatic dissemination, and induce immunoregulation. LEC phenotype and functions are strongly altered by inflammation or infections, which may directly influence on-going immune responses (4). In particular, it has been suggested that LECs immersed in the tumor microenvironment (TME) can act as immunoregulators of the anti-tumor T cell response (5). *In vitro* studies have further shown that, tumor derived LECs exhibit altered gene expression profiles compared to dermal derived LECs (6) and upregulate PD-L1 to inhibit T cell activation (7, 8). On the other hand, a recent study has suggested that tumor-associated (TA) LVs might be beneficial for the efficacy of anti-PD-1 immunotherapy (9). Therefore, depending on the stage of tumor progression and on the immunological settings (immune evasion/immunosubversion or immunotherapy), LV might display positive and/or negative effects on tumor immunity. It is thus urgent to decipher precisely the roles for LVs in tumor cell dissemination and anti-tumor T cell immunity. In this review, we discuss the ability of LECs to shape tumor development through their contribution to tumor cell spreading and regulation of anti-tumoral T cell responses.

## Lymphatic Vessels as Immunoregulators in Non-Tumor Context

LVs develop as a hierarchical vasculature facilitating a unidirectional drainage system of fluid and cells from tissues toward draining lymph nodes (LNs) (10). They interlace the blood vessel circulation and play a crucial role in lipid absorption, tissue fluid homeostasis and immunity (11). The lymphatic system is a linear and blind-ended circuit. Initial lymphatic capillaries are composed of a single layer of LECs with minimal basement membrane and are not covered by pericytes or smooth muscle cells. This particular organization of LECs is highly permeable for the uptake of cells, macromolecules and interstitial fluids (12). Lymphatic capillaries drain to collecting lymphatics defined by pericyte and smooth muscle cell coverage, continuous basement membrane with “zipper-like” junctions, and a system of valves preventing retrograde flow (12, 13). Our knowledge of multiple LV functions has quickly evolved, based on the identification of LEC markers such as the transcription factor Prox-1 and the surface protein LYVE-1, that are not expressed by others endothelial cells. Prox-1 is primordial for the development and the maintenance of LECs (14–16). LYVE-1 is enriched in lymphatic junctions, highly expressed in initial lymphatics, but mostly absent from LV collectors [reviewed in (12)]. This molecule is implicated in dendritic cells (DCs) trafficking within LVs (17). LECs also express GP38 (podoplanin) and platelet endothelial cell adhesion molecule (PECAM-1 or CD31) that are markers shared with fibroblastic reticular cells (FRCs) and blood endothelial cells (BECs), respectively. An important function of lymphatics is to transport immune cells from peripheral tissues to LNs and therefore to participate to immune response initiation (18–21). Transcriptomic analysis of *ex vivo* LN stromal cell (LNSC) subsets in distinct immunological situations established that FRCs, BECs, and LECs express a multitude of immune mediators and growth factors that may influence the immune system. LNSCs are strongly modulated by inflammation or infections, and may contribute as active participants of on-going immune responses. In addition, a more precise characterization of these cells within distinct conditions suggested that LNSCs are specialized for their unique microenvironment (4). This might reflect a functional specialization of LNSC subsets depending on the organ microenvironment. Apart from their effect on tissue drainage and immune cell migration, LECs regulate T cell responses through different mechanisms (22). First, different studies in mice showed that steady-state LN LECs participate to peripheral T cell tolerance by presenting endogenously expressed tissue-restricted antigens (17, 18) through MHC class I (MHCI) molecules and eliminating autoreactive CD8^+^ T cells (23–25). LN LECs can also cross-present exogenous antigens onto MHCI molecules, and further drive the apoptosis of antigen-specific CD8^+^ T cells (26). Whether LN LECs have an impact on peripheral CD4^+^ T cell responses in different immunological settings remains largely unknown and controversial. On the one hand, Rouhani et al. showed that LECs were unable to load MHC class II (MHCII) molecules with antigenic peptides due to their lack of H2-M expression at steady-state (27). However, LECs express the promoter IV (pIV) of CIITA, the master regulator for MHCII molecule expression (28). CIITA pIV being inducible by IFN-γ (29), LECs might require exposure to IFN-γ to upregulate H-2M molecules and be capable of MHCII-restricted antigen presentation. On the other hand, we published that surface MHCII expression on LNSCs results from the combination of both endogenous and acquired molecules. *In vitro* and *in vivo*, LNSCs further present peptide-MHCII complexes acquired from DCs to CD4^+^ T cells to induce their dysfunction. In particular, LECs specifically induce CD4^+^ T cell death, whereas LECs, BECs and FRCs all induce T cell anergy (28). Moreover, our recent studies demonstrate that the loss of MHCII expression on LNSCs in murine LNs impairs peripheral CD4^+^ T cell tolerance, and alters regulatory T cell populations, resulting in signs of spontaneous autoimmunity in elderly (30). Their lack of costimulatory molecules could explain LNSC implication in T cell tolerance. By releasing the sphingosine 1-phosphate (S1P), LECs play also an important role in the egress of activated T cells from LNs (31, 32). In addition, LEC-derived S1P is involved in naïve T cells survival, its signaling further providing sufficient energy to maintain their steady-state recirculation (33). LECs are also capable of preventing T cell activation and proliferation in a negative regulatory feedback process. Indeed, LECs from LNs produce nitric oxide in response to inflammatory signals (IFN-γ and TNF) produced by T cells, inhibiting back T cell activation (34). Finally, during inflammation, LECs present in collecting LV or in the skin suppress DC maturation via a Mac-1/ICAM-1 dependent mechanism (35), or through prostacyclin synthesis, respectively (36), leading to subsequent dampening of T cell activation.

## Tumor-associated Lymphangiogenesis and Metastasis

The mortality linked to solid tumors is mainly associated with their capacity to disseminate to distant organs in a process known as metastasis (37). LVs are essential in tumor cell spreading as they function as “highways” connecting primary tumors to secondary lymphoid organs. The process of lymphatic proliferation, sprouting and enlargement during tumor progression, known as tumor lymphangiogenesis, and its implication in the spread of the disease has been studied for many years. TA-lymphangiogenesis correlates with metastasis and poor prognosis in several cancer types [as depicted in (10)], illustrating the relevance of lymphatic vasculature to cancer biology. A retrospective analysis of melanoma patients with lung metastases showed that high LV density and lymphatic invasion in metastatic regions were associated with poor prognosis (38). Moreover, LVs and immune cell infiltrates positively correlate in human metastatic cutaneous melanoma and colorectal cancer (39, 40). Therefore, therapies aiming at blocking tumor lymphangiogenesis are being considered as promising approaches for the treatment of such malignancies [as discussed in (41)]. Importantly, several inhibitors targeting distinct actors of lymphangiogenesis have been developed in murine tumor models and could be translated into clinic to reduce metastasis. Accordingly, the inhibition of the prolymphangiogenic VEGFR-3 signaling by using VEGFR-3 blocking antibodies or VEGF-C/D trap reduces LN, and/or distant organ metastasis in different tumor mouse models (42–45). Conversely, overexpression of two lymphangiogenic factors VEGF-C and VEGF-D increases metastasis dissemination to sentinel LNs (46–49). In particular, molecules or antibodies blocking VEGF-C/VEGFR3 signaling have been tested in clinical trials, some have gone one to be approved for cancer treatment (10, 50) [as reviewed in (10)]. However, although blockade of VEGFR3 has no noticeable effect on established lymphatics, VEGF-C signaling has been described to promote homeostasis of intestinal and brain meningeal LVs (51, 52). Therefore, it would be crucial to develop treatment that specifically target pro-tumorigenic LEC functions in order to exclude any potential intestinal or neurological side effect.

In mice, LECs from tumors present a distinct molecular profile compared to dermal LECs. Altered pathways include chemokines, extracellular matrix, cell adhesion, and inflammatory responses (6). These observations reflect significant levels of LEC plasticity that is highly regulated by the tissue microenvironment.

### Pro-Lymphangiogenic Factors and Tumor Cell Spreading

The TME is composed of cancer cells, the extracellular matrix (ECM), stromal cells, and various immune cell types, impacting both tumor cell development and anti-tumor immunity. All these cells produce many factors that lead to the establishment of an intratumoral environment characterized by chronic inflammation, immunosuppression, angiogenesis, and lymphangiogenesis, the latter being the focus of this review (Figure 1.1).

**Figure 1:**
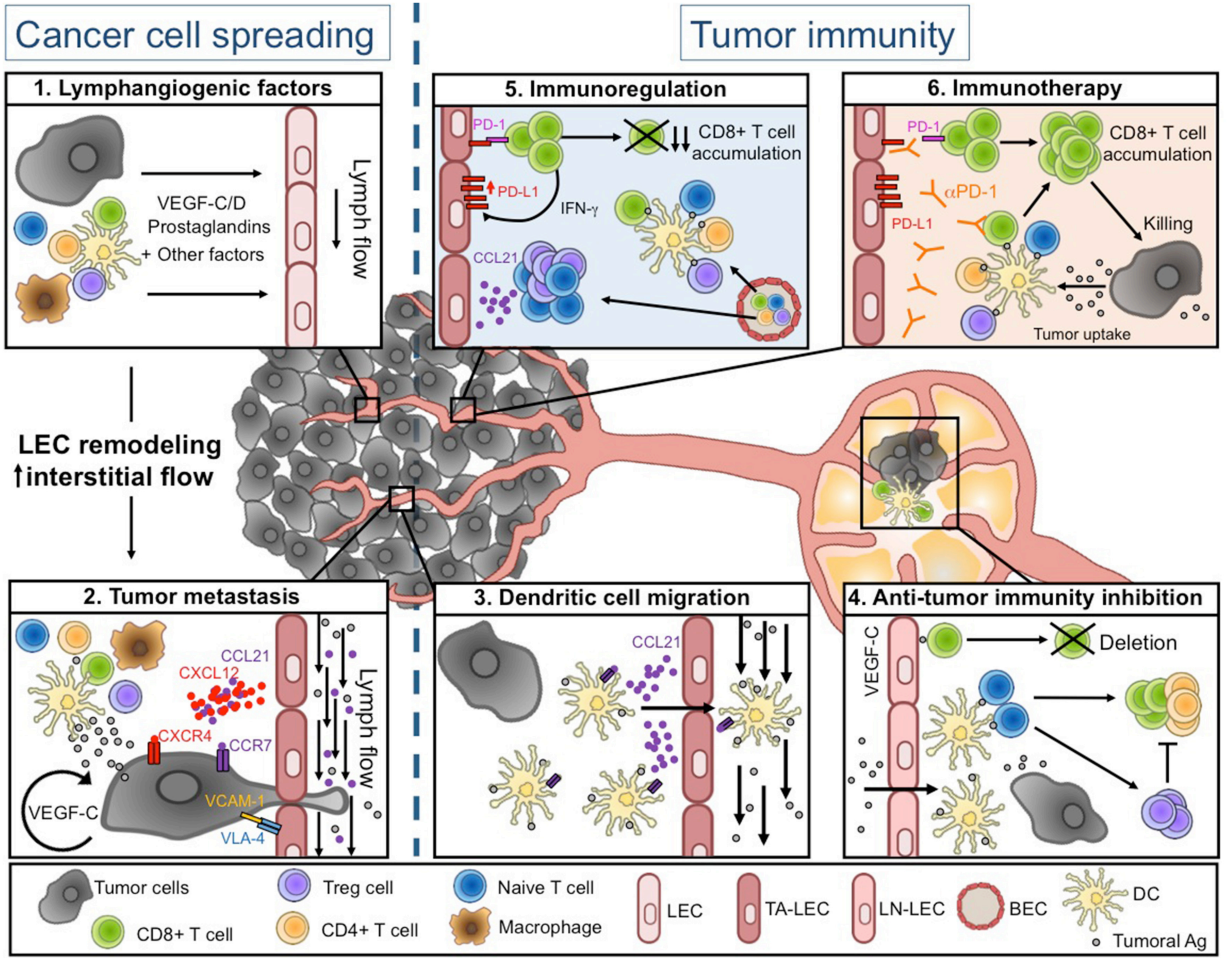
Lymphatic vessel functions during tumor progression. **(Left)** *Tumor-associated lymphatics facilitate tumor cell spreading*. Soluble factors produced in the tumor microenvironment (TME) induce LEC remodeling and interstitial flow increase **(1)**, resulting in enhanced tumor cell migration into lymphatic vessels (LVs) **(2)**. **(Right)** *Tumor-associated lymphatics regulate anti-tumor immunity*. Tumor associated (TA-) LECs actively promote DC migration toward draining lymph nodes (LNs) **(3)**. DCs further present tumor-antigens to naïve T cells, leading to initiation of adaptive anti-tumor immunity. In tumor-draining LNs, VEGF-C exposed LEC cross-present tumor-antigens (Ags) and induce the deletion of anti-tumor CD8^+^ T cells **(4)**. Intratumorally, naïve and activated T cells are weakly restimulated by local DCs, due to the immunosuppressive TME. The TME favors in particular the infiltration of Treg and naïve T cells through a CCL21-dependent pathway. TA-LECs also express high levels of PD-L1 in response to IFN-γ produced by effector T cells **(5)**. Upon anti-PD-1 immunotherapy, TA-LEC mediated immunosuppression might be abrogated, contributing to enhanced T cell activation and tumor cell elimination **(6)**. Drawing designed by Rémi Jeandenand.

The proliferation, migration and survival of LECs depend mainly on VEGFR2/3 signaling axis, which is driven by VEGF-C and VEGF-D (Vascular Endothelial Growth Factors –C and –D) (53, 54) produced by many different cell types, including tumor cells and immune cells. VEGF-C and VEGF-D, considered to be major drivers of tumor lymphangiogenesis, are associated with LN and/or distant organ metastasis (38, 46–48, 55–57) (Figure 1.1). Using orthotopic spontaneous metastasis models in nude mice, it has been shown that VEGF-C expression by tumor cells favors metastatic propagation in distal organs (57). Moreover, a recent study indicated that, in a transgenic mouse model with increased lymphangiogenesis in the lung, TA-LVs contribute to the dissemination of metastases to distant organs (38). *In vitro* studies have deciphered the molecular mechanisms implicated in the activation of VEGF-C/D signaling pathway. Following VEGFR-3 engagement, the protein kinase C is activated, leading to the phosphorylation of AKT, and subsequent LEC migration, survival and proliferation (53). Neuropilin 2 (Nrp-2), an additional receptor for VEGF-C, is also expressed by LECs, and contributes to lymphatic sprouting (58, 59).

In several human cancer, VEGF-C and COX-2 (cyclo-oxygenase 2, an enzyme implicated in prostaglandin pathway) expression are associated with LV density and LN metastasis (60–63). Interestingly, preclinical and clinical trials using different Non-Steroidal Anti-Inflammatory Drugs (NSAIDs), blocking COX-2 and subsequent prostaglandin production, have reported a decrease in cancer incidence, tumor cell dissemination, and finally global cancer morbidity. These observations suggest that NSAIDs could be applied for the treatment of metastasis (64–66). In mice, beside a direct effect on LECs, VEGF-C/D increases the levels of prostaglandins in the TME, further promoting TA-lymphangiogenesis. VEGF-D indeed inhibits the enzyme 15-hydroxyprostaglandin dehydrogenase (15-PGDH), therefore enhancing LEC exposure to prostaglandins in collecting lymphatic vessels (67). The engagement of EP3 signaling (prostaglandin E2 receptor 3) on tumor-associated stromal cells promotes lymphangiogenesis (68). Moreover, increased amounts of prostaglandins amplify the production of VEGF-C by tumor and immune cells, contributing to lymphangiogenesis and tumor cell dissemination (62, 63).

TNF-α interaction with its TNF receptor 1 (TNFR-1) triggers VEGF-C secretion by tumor-associated macrophages (TAM), amplifying LV expansion and metastasis (69). On the other hand, TNF-α signaling in LECs directly favors their proliferation and their migration, without however being sufficient to constitute a fully competent lymphatic network (69). Indeed, TNF-α induced lymphangiogenesis completely depends on the VEGF-C/VEGFR3-induced LEC tip formation. Similarly, VEGF-C/VEGFR3-induced LEC tip formation is required to trigger fibroblast growth factor (FGF2) induced lymphangiogenesis and foster tumor metastasis in mice (70). In contrast, proangiogenic factors such as platelet derived growth factor B (PDGF-BB) (71) and angiopoietins (ANGPTs) (72) can act as direct lymphaniogenic factors by binding, respectively, PDGF-BB receptors and receptors Tie1/2 expressed by LECs. Whether TGF-β enhances (73, 74) or inhibits (75) lymphangiogenesis depends on tumor models, rendering difficult the targeting of this cytokine for the regulation of LEC remodeling in tumors.

### Mechanism of Metastasis Dissemination

Emerging evidence suggest that lymphatic vessels undergo several changes in response to lymphangiogenic factors during the course of metastasis. In addition to promoting tumoral cell transportation, LVs deliver lymphangiogenic factors produced by the primary tumor to condition sentinel LNs prior to the arrival and seeding of cancer cells (56, 76–78). In the mouse B16F10 melanoma model, LVs from distant metastatic regions, such as LNs and lungs, attract chemoresistant CD133^+^CXCR4^+^ melanoma cells by secreting CXCL12 (79).

TA-LVs were for long described as passive conduits for tumoral cell spread toward sentinel LNs and distant organs. However, several studies highlighted that the tumor microenvironment actively modifies LV features of primary tumor and draining LNs to further promote metastasis. VEGF-C acts in an autocrine manner to improve metastasis dissemination by favoring proteolytic activity and motility of tumor cells (80). Besides its direct effect on tumoral cells, VEGF-C modulates the expression of integrins and chemokines by LECs to facilitate tumor invasiveness. The integrin α4β1 (or VLA-4), which is considered as a marker of activated and proliferating LECs in human and murine tumors (81), is activated by the VEGF-C/PI3Kα pathway in LECs to promote lymphangiogenesis and tumor metastasis in LNs. Therefore, the blockade or the genetic deletion of this integrin on LVs prevents LEC migration and invasion, and inhibits VCAM-1 mediated adhesion of tumoral cell to LECs (82). The secretion of CCL21 by LECs, which drives CCR7-dependent tumor migration through LVs, is also enhanced in response to VEGF-C (80). Moreover, CCL21-dependent recruitment of innate lymphoid cells results in the production of CXCL13 by tumoral stromal cells, which in turn induces metastasis through RANK/RANKL signaling (83). Transmural flow modulates LEC function by promoting the expression of CCL21 and by downregulating VE-cadherin and PECAM-1, two adhesion molecules crucial for cellular junctions (84). Modification of interstitial flow influences CCR7 ligand secretion by tumoral cells, providing an autologous chemotactic gradient (85). In human, CCR7 expression by tumor cells is associated with LN metastasis in several cancers (86–88) (Figure 1.2).

CCL1 secretion by LECs located in the subcapsular sinuses of LNs is crucial to control tumor cell invasion into LNs. Indeed, blocking the CCL1 receptor (CCR8) inhibits metastasis by preventing tumor cell egress from collecting lymphatics into LNs without affecting their entry into intratumoral lymphatics (89). Recently, the screening of 810 mutant mouse strains allowed the identification of 23 genes that, when disrupted, alter the establishment of metastatic foci (90). Notably, they demonstrated that the deletion of the sphingosine-1-phosphate transporter SPNS2 in LECs decreases pulmonary metastasis and promotes effector T cell and natural killer cell infiltration in lungs (90).

Recently, Black et al., have shown that the pro-lymphangiogenic factor COX-2 enhances the expression of semaphorin 7a (sema7a) in breast tumoral cells. This leads in turn to the activation of β1-integrin receptors on adjacent tumoral cells and LECs, to finally increase lymphangiogenesis and cancer cell dissemination (91). Moreover, sema7a induces gp38 upregulation by tumor-infiltrating macrophages, therefore promoting their adhesion to LVs and consequently boosting lymphangiogenesis and metastasis in breast cancer (92). In agreement, Sema7a gene expression is observed in a high frequency in human breast cancer and correlates with metastasis and poor prognosis (91).

Apart from their implication in metastasis dissemination, accumulating studies indicate that LECs modulate anti-tumor immunity. The roles of LECs in tumor spreading and anti-tumoral immune responses are discussed below.

## Dual Role of Tumor-associated Lymphatic Vessels in Anti-tumor Immunity

Growing evidence highlight that, in addition to acting as drains for soluble factors and tumoral cell transport, TA-LVs further play important roles in shaping antitumor immunity. Therefore, the modulation of lymphangiogenesis could impact not only metastasis dissemination but also anti-tumor immunity and primary tumor growth. In the context of solid tumors, lymph flow from tumors is increased, driving intense interstitial flow in the tumor stroma, and enhancing lymphatic drainage to the draining LNs (93). TA-LVs are primarily required for the recruitment of immune cells and adaptive immune response initiation (39, 94). However, immunosuppressive features of LECs in TME will subsequently dampen ongoing anti-tumor immunity (5). Therefore, LVs play a dual role on tumor immunity that might be temporally regulated. Finally, immunotherapy approaches can be potentialized by TA-lymphangiogenesis in melanoma tumors (9), further highlighting the relevance of modulating LV functions during tumor development.

### Lymphatic Vessels Are Necessary for the Initiation of Anti-Tumoral Responses

T cell activation and infiltration in tumors are key steps of antitumor immunity. Indeed, while Treg infiltration is associated with a poor outcome in patients, intratumoral cytotoxic T lymphocytes are beneficial for clinical outcome (95, 96). Although some studies have suggested that naïve T cell could infiltrate tumors and be locally activated (97–99), antigen transport by dendritic cells (DCs) through LVs toward draining LNs is nevertheless crucial for the initiation of tumor-specific T cell responses, at least in melanomas (39, 100). Indeed, tumor drainage, DC trafficking and subsequent induction of anti-tumor adaptive immune responses are drastically impaired in transgenic mice lacking or with disturbed local LVs (39, 94). Upon inflammation, LECs in afferent LVs produce CCL21 that is necessary to DC egress from the tissue toward lymphatics (101, 102). Moreover, the expression of CLEC-2 by DCs is essential for their migration into LNs. The activation of CLEC-2 by GP38, which is highly expressed by LECs and FRCs, induces actin polymerization and motility of DCs (103). In a tumoral context, CCR7 expression by DCs is primordial for their migration into tumor draining LNs and subsequent T cell activation (100) (Figure 1.3).

In agreement with a role for lymphatic vasculature in the initiation of anti-tumor immunity, lymphatic vessel density (LVD) or lymphatic gene expression in primary tumors of colorectal or melanomas patients positively correlates with inflammation and immune cell infiltration (9, 39, 40, 104).

### Lymphatic Vessels Suppress Effector T Cells During Tumor Progression

The lymphangiogenic factor VEGF-C produced in the tumor favors immunological tolerance in murine melanoma, including the induction of tumor-specific CD8^+^ T cell deletion (5) (Figure 1.4) (5, 26). This is consistent with studies in human melanoma, where active CTLs can be found in the circulation, while they exhibit an exhausted phenotype when localized in tumors (105). In addition, LECs in tumor draining LNs cross-present tumor antigens through MHCI complexes, and further drove the apoptosis of tumor-specific CD8^+^ T cells. The expression of the immunosuppressive molecule PD-L1 is enhanced at the surface of LECs after antigen specific interaction with CD8^+^ T cells *in vitro* (7, 26). Moreover, blockade of PD-L1 on antigen pulsed immortalized LECs *in vitro* increases CD8^+^ T cell activation (7). *In vivo*, in several tumor mouse models, TA-LECs express higher levels of PD-L1 compared to naïve skin LECs (7, 8), the highest PD-L1 expression being observed in immunogenic tumors (8). Recently, Lane et al. demonstrated that PD-L1 expression by non-hematopoietic cells prevents CD8^+^ T cells accumulation in melanoma. IFN-γ production by antigen-specific CD8^+^ T cells is primarily necessary to induce PD-L1 expression on LECs. Using mice with LECs deficient for IFN-γ receptor, they established that the specific loss of IFN-γ sensitivity in LVs improves CD8^+^ T cell-dependent control of melanoma tumor growth and mouse survival (8). Thus, during tumor development, a negative feedback loop is set up between LECs and T cells. LECs up-regulate PD-L1 expression in response to IFN-γ produced by tumor specific CD8^+^ T cells, and subsequently inhibit T cell accumulation in tumors (Figure 1.5).

In human metastatic melanoma, VEGF-C expression positively correlates with T cell infiltration and CCL21 expression (9). CCL21 plays a crucial role in the establishment of a tolerogenic tumor microenvironment by recruiting CCR7^+^ regulatory T cells in primary tumors and by promoting the formation of lymphoid like stromal structures with immunosuppressive features (106). CCL21 further attracts naïve T cells that can be locally activated in response to immune blockade or vaccination (9).

## Concluding Remarks

Recent studies indicate that tumor-associated LECs significantly contribute to shaping the immunosuppressive TME, therefore helping tumors hijack the immune system from an efficient to an incompetent anti-tumor response. Altogether, several observations highlight a new role for lymphatics in promoting tumor development, suggesting that lymphatic endothelium in the local microenvironment may be a novel target for immunomodulation. In agreement with these hypotheses, a recent publication demonstrated that following exposure to tumor derived factors, FRCs of the tumor draining LNs undergo multiple changes to convert into a immunosuppressive phenotype, such as decreased production of IL-7 and CCL19/21 (107). Whether a similar profound reprogramming occurs to LECs in tumor draining LNs remains to be determined.

Whereas, VEGFC driven TA-lymphangiogenesis correlates with increased intratumoral inflammation (39) and immune suppression in progressing tumors (5), it seems also to be necessary for the response of the tumor microenvironment to immunotherapeutic intervention, as demonstrated for PD-1 blocking antibodies (9) (Figure 1.6). This suggests that TA-LECs potentiate immunotherapy by attracting naive T cells through a CCL21 dependent mechanism. Accordingly, LVs and immune cell infiltrates positively correlated in metastatic cutaneous melanoma and colorectal cancer patients (39, 40). Once in the tumor, naive T cells can be locally primed upon PD-1 blockade, which reverts the immunosuppressive T cell imprinting and induces long-lasting anti-tumor immunity. Therefore, it is tempting to speculate that LV density in tumors could be used as a predictor for positive response to immune checkpoint blockade. Additional research will determine how to selectively target LEC immunosuppressive functions in tumors, which could, combined to immunotherapeutic approaches, lead to the conversion of a “cold” into “hot” immunogenic TME and potentiate anti-tumor T cell responses.

## Author Contributions

LG, A-OG, and SH wrote the manuscript. LG conceptualized Figure 1.

### Conflict of Interest Statement

The authors declare that the research was conducted in the absence of any commercial or financial relationships that could be construed as a potential conflict of interest.
